# Interaction Between CD34^+^ Fibrocytes and Airway Smooth Muscle Promotes IL-8 Production and Akt/PRAS40/mTOR Signaling in Asthma

**DOI:** 10.3389/fmed.2022.823994

**Published:** 2022-04-25

**Authors:** Ting-Yu Lin, Po-Jui Chang, Chun-Yu Lo, Yu-Lun Lo, Chih-Teng Yu, Shu-Min Lin, Chih-His Scott Kuo, Horng-Chyuan Lin

**Affiliations:** ^1^Department of Thoracic Medicine, Chang Gung Memorial Hospital, Taipei, Taiwan; ^2^College of Medicine, Chang Gung University, Taoyuan, Taiwan

**Keywords:** fibrocytes, airway smooth muscle cells, interleukin-8, interleukin-33, PRAS40, mTOR, AKT, asthma

## Abstract

**Background:**

The circulating progenitor cells of fibroblasts (fibrocytes) have been shown to infiltrate the airway smooth muscle compartment of asthma patients; however, the pathological significance of this discovery has yet to be elucidated. This study established a co-culture model of airway smooth muscle cells (ASMCs) and fibrocytes from asthmatic or normal subjects to evaluate innate cytokine production, corticosteroid responses, and signaling in ASMCs.

**Methods:**

CD34^+^ fibrocytes were purified from peripheral blood of asthmatic (Global Initiative for Asthma treatment step 4–5) and normal subjects and cultured for 5∼7 days. In a transwell plate, ASMCs were co-cultured with fibrocytes at a ratio of 2:1, ASMCs were cultured alone (control condition), and fibrocytes were cultured alone for 48 h. Measurements were obtained of interleukin-8 (IL-8), IL-6, IL-17, thymic stromal lymphopoietin, and IL-33 levels in the supernatant and IL-33 levels in the cell lysate of the co-culture. Screening for intracellular signaling in the ASMCs after stimulation was performed using condition medium from the patients’ co-culture (PtCM) or IL-8. mRNA and western blot analysis were used to analyze AKT/mTOR signaling in ASMCs stimulated via treatment with PtCM or IL-8.

**Results:**

Compared with ASMCs cultured alone, IL-8 levels in the supernatant and IL-33 levels in the ASMCs lysate were significantly higher in samples co-cultured from asthmatics, but not in those co-cultured from normal subjects. Corticosteroid-induced suppression of IL-8 production was less pronounced in ASMCs co-cultured with fibrocytes from asthma patients than in ASMCs co-cultured from normal subjects. ASMCs stimulated using PtCM and IL-8 presented elevating activated AKT substrate PRAS40. Treatment with IL-8 and PtCM increased mRNA expression of mTOR and P70S6 kinases in ASMCs. Treatment with IL-8 and PtCM also significantly increased phosphorylation of AKT and mTOR subtract S6 ribosomal protein in ASMCs.

**Conclusion:**

The interaction between ASMCs and fibrocytes from asthmatic patients was shown to increase IL-8 and IL-33 production and promote AKT/mTOR signaling in ASMCs. IL-8 production in the co-culture from asthmatic patients was less affected by corticosteroid than was that in the co-culture from normal subjects. Our results elucidate the novel role of fibrocytes and ASMCs in the pathogenesis of asthma.

## Background

In asthma patients, immune cells have been shown to influence the contractic and proliferative functions of airway smooth muscle cells (ASMCs) (1, 2). Infiltration of the ASM layer of asthmatic patients by an elevated number of mast cells (3) has been shown to promote ASMC differentiation into its contractile phenotype (4). CD4^+^ T cells within the ASM layer have also been shown to promote ASMC proliferation (5, 6). ASMCs are the source of inflammatory mediators perpetuating chronic inflammation (7). An increase in ASM mass is a feature of asthmatic airways and the cellular mechanism underlying ASMC proliferation, such as phosphatidylinositol 3-kinase or AKT-mammalian target of rapamycin (mTOR) (8, 9).

One study reported on the migration and infiltration of CD34^+^ progenitor cells into the ASM compartment in asthmatic patients (10). These circulating progenitor cells, referred to as fibrocytes, co-express hematopoietic stem cell marker CD34, collagen 1, and panhematopoietic marker CD45, but do not express CD14 (11, 12). In CD34^+^ cells from the blood of asthmatic patients, fibrocytes have been shown to produce proinflammatory and profibrotic mediators under type 2 cytokine stimulation (13). Nonetheless, few studies have addressed the pathological significance of fibrocytes infiltration in the ASM layer. Two studies demonstrated that fibrocytes promoted inflammatory cytokine production and gel contraction when co-cultured with ASMCs. However, fibrocytes did not affect the proliferation or apoptosis of ASMCs (14, 15). A number of questions still need to be addressed. For example, do the suppressive effects of corticosteroids differ between asthmatic patients and normal subjects? Do fibrocytes affect upstream proliferative signaling in ASMCs, despite previous reports that fibrocytes did not affect the proliferation of ASMCs in terms of Ki67 or cell size?

In the current study, we addressed these questions by establishing a co-culture model by which to evaluate the interactions between ASMCs and CD34^+^ fibrocytes, respectively, from asthmatic patients and normal subjects. We analyzed the asthma-related expression of innate inflammatory cytokines and the suppressive effects of corticosteroids. Array screening of active signaling in ASMCs revealed that the AKT-mTOR pathway may be activated when co-cultured with asthmatic fibrocytes.

## Materials and Methods

### Subjects

Thirty asthma patients and ten normal subjects were recruited (Table 1). The asthma patients presented a consistent history including one or more of the following: (1) >12% improvement in forced expiratory volume in 1 s (FEV1) after administration of bronchodilator; (2) PC20 of methacholine test <8 mg/mL; (3) Diurnal variation of peak expiratory flow >20%. Patients in the current study were undergoing treatment in accordance with 2016 Global Initiative for Asthma (GINA) guidelines levels 4–5. Subjects were non-smokers or ex-smokers with a previous smoking history of <10 pack-years and free from respiratory or systemic infections in the previous 2 months. All subjects provided informed consent and the study protocol was approval by the Chang Gung Medical Foundation Institutional Review Board (No. 103-6844A3).

**TABLE 1:** Characteristics of recruited subjects.

	Normal subjects (*n* = 10)	Asthmatic patients (*n* = 30, GINA step 4, *n* = 23, step 5, *n* = 7)
Male, *n* (%)	4 (40.0)	20 (60.0)
Age, year	35.4 ± 4.6	58.6 ± 12.0****
Atopy, *n* (%)	2 (20.0)	16 (53.2)
Body mass index (kg/m^2^)	22.2 ± 2.6	25.8 ± 4.2*
Prebronchodilator FVC, liter	3.1 ± 0.6	2.2 ± 0.7***
Prebronchodilator FVC,% of prediction	91.4 ± 3.1	74.2 ± 19.9*
Prebronchodilator FEV1, liter	2.5 ± 0.5	1.6 ± 0.5****
Prebronchodilator FEV1,% of prediction	89.4 ± 6.3	65.7 ± 21.9**
FEV1/FVC,%	80.7 ± 3.4	74.6 ± 13.8
Treatment with inhaled corticosteroid and long-acting bronchodilators, *n*	0	30 (100.0)****
Treatment with oral corticosteroid, *n*, (%)	0	3 (10.0)
Treatment with biological agent, *n*, (%)	0	2 (6.7)

Data are expressed as means ± SEM or number of subjects with percentage (*p < 0.05, **p < 0.01, ***p < 0.001, ****p < 0.0001). FVC, forced vital capacity; FEV1, forced expiratory volume in 1 s.

### Isolation of CD34^+^ Fibrocytes From Peripheral Blood Mononuclear Cells

**FIGURE 1:**
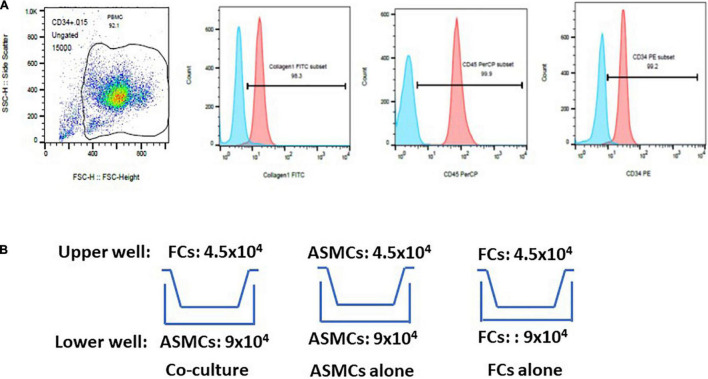
Purification of fibrocytes (FCs) after culturing cells for 5–7 days. **(A)** Cells underwent triple positive staining (> 90% positive expression) for collagen-I, CD34 and CD45 via flow cytometry. The proportion of positive expression was defined by isotype staining. **(B)** Co-culture model of airway smooth muscle cells (ASMCs) with FCs. Co-culture: ASMCs in lower well and FCs in upper well were cultured at a cell ratio of 2:1 for 48 h. ASMCs alone were cultured in the lower and upper well at a cell ratio of 2:1 as a major control. FCs alone were cultured in the same way. Culture supernatant and cell lysates was collected from lower wells for analysis.

Fibrocytes were separated in accordance with published methods with slight modifications (13, 16, 17). Briefly, peripheral blood mononuclear cells (PBMCs) were isolated from whole blood using Ficoll-Paque (GE Healthcare, Bio-science AB, Uppsala, Sweden) density gradient centrifugation. Mononuclear cells were harvested, washed, and resuspended in Iscove’s modified Dulbecco’s medium. Magnetic positive selection of CD34 was performed sequentially in accordance with manufacturer instructions (Miltenyi Biotec, Auburn, CA). Monocytes/macrophages were further depleted by negative selection of CD14. Cells isolated via immunomagnetic selection were cultured with DMEM containing 30% fibrocytes (FCS), 1% BSA, and stem cell factor (50 ng/mL). Non-adherent cells were removed within 48 h and adherent cells were cultured for an additional 5–7 days. The purity (> 90%) of fibrocytes was determined via immunofluorescence staining for CD34, CD45, and Col-I via flow cytometry (Figure 1A).

### Culturing Airway Smooth Muscle Cells

ASMCs from normal subjects were purchased from Lonza Inc. ASMCs were maintained in DMEM with 10% FBS with 5% CO_2_ at 37^°^C. Cells at the fourth to seventh passage were utilized in subsequent experiments. Information about the ASMCs donors were available in Supplementary Material.

### Transwell Culturing of Airway Smooth Muscle Cells and Fibrocytes

ASMCs and FCs from patients or normal subjects were serum-starved overnight. Cells were cultured in a transwell plate (24-well culture plate; 0.4 μm pore size; Corning, Lowell, MA) (Figure 1B). Co-culturing was conducted over a period of 48 h with 9.0 × 10^4^ ASMCs in the lower well and 4.5 × 10^4^ FCs in the upper well (i.e., cell ratio of 2:1). Note that this setup is hereafter referred to as the co-culture, which was meant to elucidate the interaction between ASMCs and FCs. We also cultured ASMCs alone in both the upper and lower wells using the same cell number ratio as a control, (hereafter referred to as ASMCs alone). We also cultured FCs alone in both the upper and lower wells using the same cell number ratio, (hereafter referred to as FCs alone). If the cultured fibrocytes were not adequate for preplanned cell numbers for co-culture, the co-culture will be processed by adjusting cell numbers with fixed ratio of lower and upper well of co-culture to 2:1. Supernatant from the cultures was assayed for interleukin-8 (IL-8), IL-6, IL-17, thymic stromal lymphopoietin (TSLP), and IL-33 using commercial ELISA kits (R&D systems and Boster Biological technology for TSLP). Cell lysate from the lower wells was also collected and assayed for IL-33 levels via ELISA. The sensitivity limit for IL-8, IL-6, IL-17, TSLP, and IL-33 was 7.5, 0.7, 15, 10, and 1.65 pg/ml, respectively. The ELISA data was presented by percentage of control. Condition medium was collected for further experiments.

### Corticosteroid Suppression of Cytokine Production in Co-culture of Airway Smooth Muscle Cells and Fibrocytes

ASMCs were co-cultured with fibrocytes of asthmatic patients or normal subjects at a ratio of 2:1 in starving medium with serial doses of dexamethasone (Sigma-Aldrich; 10^–4^ to 10^–10^ M) or vehicle (DMSO) for 48 h. IL-8/IL-6 levels in the supernatant and IL-33 levels in the cell lysate from ASMCs were assessed via ELISA.

### Path Scan Intracellular Signaling Array

An intracellular signaling array was used to detect the effects of IL-8 and co-culturing with fibrocytes on intracellular signaling in ASMCs. Briefly, 80% confluent ASMCs were cultured for 24 h in a 24-well plate with IL-8 (20 ng/ml) or condition medium from patient co-culture (PtCM). Cells were then harvested and lysed for 5 min with ice-cold cell lysis buffer containing a cocktail of protease inhibitors. The lysates were assayed for intracellular signaling molecules using a PathScan^®^ Intracellular Signaling Array kit (#7323; Cell Signaling Technology). The intensity of chemiluminescent signals was quantified using a UVP biospectrum 810 imaging system (Upland, CA, United States) in accordance with the protocol outlined by the manufacturer.

### Effect of Interleukin-8 and Condition Medium on AKT/Mammalian Target of Rapamycin Signaling and Cytokine Production by Airway Smooth Muscle Cells

Due to the limited number cells in the co-culture experiment, we were unable to obtain enough ASMCs for real-time reverse transcription or western blot analysis. Thus, we cultured fresh ASMCs in the 6-well culture plate with 80% confluence to obtain cells for this experiment. PtCM was diluted with DMEM medium at a ratio of 1:3 for culturing in 6-well plate. ASMCs were then subjected to qPCR or western blot analysis after stimulation using IL-8 or PtCM.

### Quantitative Real-Time Reverse Transcription

Over period of 0, 1, 2, 6, and 24 h, ASMC expression was stimulated using IL-8 (20 ng/ml) or PtCM or ASMC culture medium alone or fibrocyte culture alone for 2 h. PBS treatment as the control. Samples that underwent PBS stimulation were used as a control. Total RNA was isolated from co-cultured ASMCs using TRIzol reagent (Invitrogen, Grand Island, NY) in accordance with the manufacturer’s instructions. cDNA was reverse-transcribed from isolated RNA by incubating 1 μg of DNase-treated RNA using the HiscriptI First Strand cDNA synthesis kit (BioNovus Life Sciences). qPCR was performed using TOOLS SuperFast SYBR Qpcr Reagent (Biotools) in a LightCycler 2.0 System (Roche Applied Science). Sequences of mTOR, P70S6 kinase (PS6K), and 18s rRNA are described in Table 2. Samples were denatured at 95^°^C for 1 min, followed by 50 cycles of annealing and extension at 95^°^C for 10 s, 60^°^C for 20 s, and 72^°^C for 20 s. All qPCR results were normalized to the 18s rRNA of the internal control. Data were obtained using the 2-ΔΔCt method using LightCycler software.

**TABLE 2:** Primers used for real-time RT-PCR.

Gene symbol	Sequence	Amplicon length (bp)
PS6K-Forward (F)	5′-AAgggggCTATggAAAggCAA-3′	21
PS6K-Reverse (R)	5′-AATCCACgATgAAgggATgCT-3′	21
mTOR-F	5′-gAACCTCAgggCAAgATgCT-3′	20
mTOR-R	5′-CTggTTTCCTCATTCCggCT-3′	20
18s rRNA F	5′-CTTAgAgggACAAgTggCg-3′	19
18s rRNA R	5′-AgCCTgAgCCAgTCAgTgTA-3′	20

### Western Blot Analysis

After treatment using IL-8 or PtCM followed by electrophoresis, proteins from ASMCs were transferred to nitrocellulose membranes, which were blocked with 0.1% Tween 20 in Tris-buffered saline with 5% non-fat dry milk, washed, and incubated overnight with primary antibodies. Due to the limited quantity of PtCM, we selected antibodies specific to Akt, Ser473 phospho-Akt, PARS40, Thr246 phospho-PARS40, S6 ribosomal protein and phospho-S6 ribosomal protein (Cell Signaling), and beta-actin (Millipore). Following incubation with appropriate secondary antibodies, the proteins of interest were visualized via Enhanced Chemiluminescent Assay (ECL) using horseradish peroxidase. Relevant band intensities were quantified and analyzed using a UVP ChemStudio PLUS Touch scanner (Analytik Jena AG, Jena, Germany) in conjunction with VisionWorks software (Analytik Jena AG, Jena, Germany) and Image Gauge Ver 4.0, Science Lab (FUJIFILM, Tokyo, Japan).

### Statistical Analysis

Data were expressed as mean ± S.E. An unpaired two-tailed Student’s *t*-test was used for single comparisons. The Chi-square test or Fisher’s exact test was used to compare categorical data in cases where the sample size was small. Cytokine, mRNA, and protein levels in co-cultures were expressed in terms of fold change compared to levels in the ASMC culture alone. One way ANOVA was used with Tukey’s test to compare the effect of co-culture or IL-8 stimulation with results obtained from the control group. Probability values of < 0.05 were considered significant.

## Results

### Co-culturing of Airway Smooth Muscle Cells and Fibrocytes From Asthmatic Patients Increased Interleukin-8 Expression in Supernatant and Interleukin-33 Expression in Airway Smooth Muscle Cells, Compared to Culturing Airway Smooth Muscle Cells Alone

**FIGURE 2:**
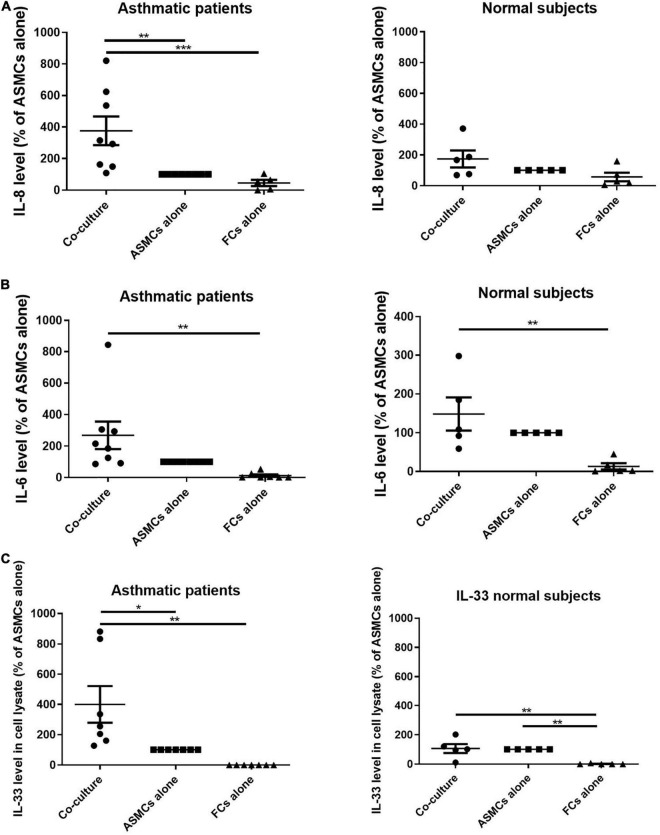
**(A)** IL-8 levels and **(B)** IL-6 levels in the supernatant and **(C)** IL-33 levels in the cell lysates from the lower well in transwell experiments involving the co-culturing of ASMCs with fibrocytes, culturing ASMCs alone, and culturing fibrocytes alone. Fibrocytes were isolated from asthmatic patients or normal subjects. Cells were cultured at the same density in each well for 48 h and cytokine levels were detected using ELISA. The level of cytokines is presented as the% of ASMCs alone. *p* < 0.001, One-way ANOVA with Tukey’s multiple comparison test (*n* = 5∼8).

ELISA was used to analyze the supernatant from co-culturing ASMCs and fibrocytes, culturing ASMCs alone, or culturing fibrocytes alone for IL-8, IL-6, IL-17, TSLP, and IL-33 production. Culturing ASMCs with fibrocytes from asthmatic patients significantly increased IL-8 levels (Figure 2A), compared to culturing ASMCs alone. Note that culturing ASMCs with fibrocytes from normal subjects had no effect on IL-8 production. Co-culturing ASMCs with fibrocytes from asthmatic or normal subjects had no effect on IL-6 levels production, compared to culturing ASMCs alone (Figure 2B). The majority of IL-6 and IL-33 of “FC alone” were below the lowest detectable concentration. We failed to detect IL-17, TSLP, or IL-33 in the supernatant of any samples. We also checked IL-33 levels in ASMCs, due to its common accumulation in cells (18). Culturing ASMCs with fibrocytes from asthmatic patients was shown to increase the intracellular level of IL-33 in ASMCs, compared to culturing ASMCs alone (Figure 2C). Culturing ASMCs with fibrocytes from normal subjects had no effect on IL-33 levels.

### Corticosteroid-Induced Suppression of Interleukin-8 Production Was More Pronounced in Samples Co-cultured With Fibrocytes From Normal Subjects Than in Samples Co-cultured From Asthmatic Patients

**FIGURE 3:**
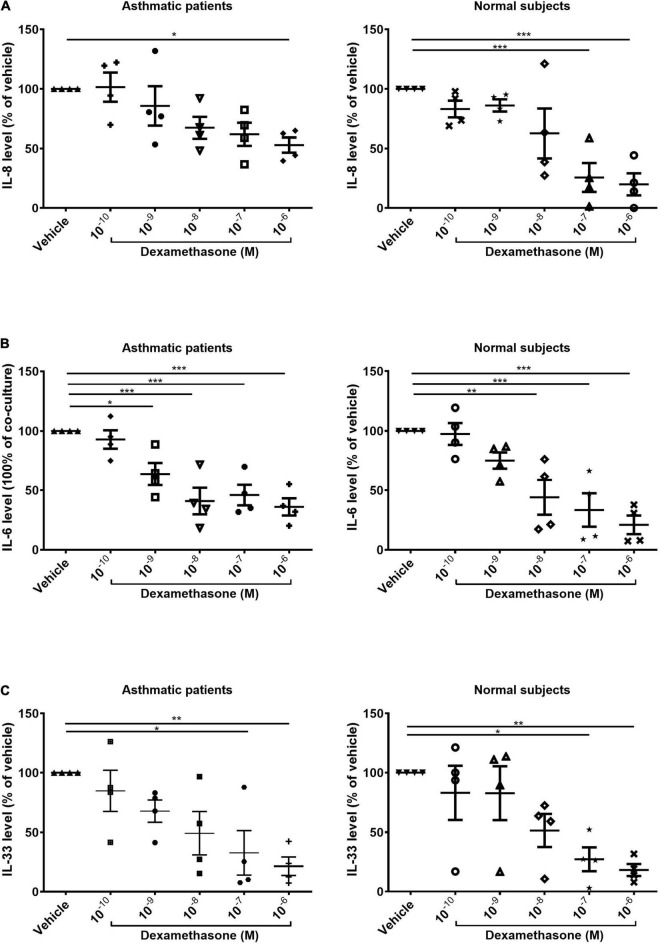
Dexamethasone-induced suppression of **(A)** IL-8 and **(B)** IL-6 in the supernatant and **(C)** suppression of IL-33 in the cell lysates of ASMCs co-cultured with fibrocytes from asthmatic patients and normal subjects (10^–10^ to 10^–6^ mol/L, 48 h). Vehicle: DMSO of same concentration in dexamethasone solution (*n* = 4). *P < 0.05; **P < 0.01; ***P < 0.001.

We also examined the expression of IL-8 after serial dosing with dexamethasone for 48 h. In samples co-cultured with fibrocytes from normal subjects, the production of IL-8 was significantly lower when dexamethasone was administered at high concentrations (10^–7^ M and 10^–6^ M, *p* < 0.001). In samples co-cultured with fibrocytes from asthmatic patients, we observed a reduction in the production of IL-8 only when dexamethasone was administered at the highest concentration (10^–6^M, *p* < 0.05), thereby by indicating less corticosteroid suppression on IL-8 production of co-culture from asthma patients (Figure 3A). Dexamethasone had a similar effect in suppressing IL-33 in ASMCs and IL-6 in the supernatant in samples co-cultured with fibrocytes from asthmatic patients as well as normal subjects (Figures 3B,C).

### Stimulation *via* Interleukin-8 or Patients’ Co-culture Increased the Phosphorylation of Proline-Rich AKT Substrate 40 kDa (PRAS40) in Airway Smooth Muscle Cells Beyond That of PBS Stimulation (Control)

**FIGURE 4:**
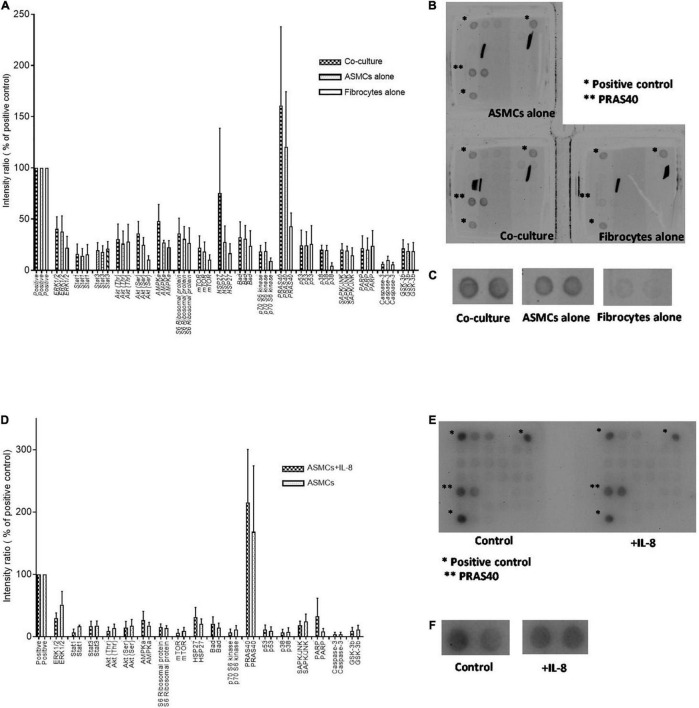
**(A)** Intracellular signaling activation in ASMCs after 24-h stimulation by condition medium from co-culturing ASMCs and fibrocytes from asthmatic patients, culturing ASMCs alone, or culturing fibrocytes alone; (*n* = 3). **(B)** The density of all signaling in ASMCs detected by array. Each signaling was done by duplication. *: positive control. ^**^: PRAS40. **(C)** The enlarged picture of activation of PRAS40 in ASMCs stimulated with condition medium of co-culture, culturing ASMCs alone or culturing fibrocytes alone. The expression of PRAS40 in ASMCs treated with co-cultured condition medium was higher than that of the positive control. **(D)** Intracellular signaling activation in ASMCs after stimulation by IL-8 (*n* = 3). **(E)** The density of all signaling in ASMCs detected by array. Each signaling was done by duplication. *: positive control. ^**^: PRAS40. **(F)** The enlarged picture of activation of PRAS40 in ASMCs stimulated with or without IL-8. Similarly, we observed relatively high PRAS40 phosphorylation in ASMCs after IL-8 stimulation.

Our results prompted analysis of the effects of PtCM and IL-8 on the intracellular signaling of ASMCs. We screened the phosphorylation status of signaling pathways in ASMCs after incubation with PtCM, medium of ASMCs, or FC culture alone for 24 h (Figures 4A–C). We observed no significantly differences between any of the samples in terms of phosphorylation; however, we observed elevate expression levels of PRAS40 in ASMCs treated with PtCM, compared to the positive control. This prompted further screening of ASMCs after stimulation by IL-8 (Figures 4D–F), which also exhibited high phosphorylation of PRAS40.

Note that PRAS40 is an AKT substrate known for the encoding of a key molecule connecting AKT signaling and mTOR activation (19, 20). Thus, our primary concern in subsequent experiments was the effects of IL-8 and PtCM on AKT-mTOR signaling in ASMCs.

### mRNA Levels of Mammalian Target of Rapamycin and PS6K in Airway Smooth Muscle Cells Increased After Stimulation Using Patients’ Co-culture or Interleukin-8

**FIGURE 5:**
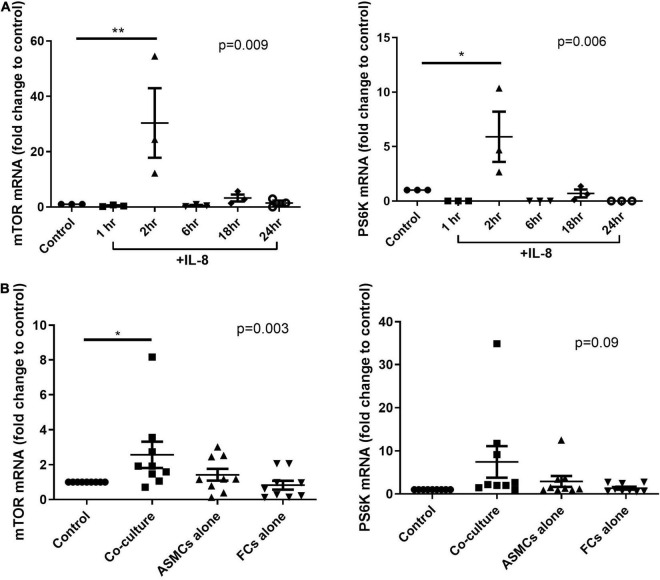
mRNA expression of mTOR or PS6K in ASMCs after **(A)** IL-8 stimulation for 1, 2, 6, 18, and 24 h or **(B)** treatment with diluted condition medium co-cultured with fibrocytes from asthmatic patients, ASMCs cultured alone, or fibrocytes cultured alone for 2 h. PBS as the control. *p < 0.05, One-way ANOVA with Tukey’s multiple comparison test (*n* = 3∼9). **P < 0.01.

We first tested the mRNA expression of mTOR and its substrate PS6K in ASMCs. The mRNA expression of mTOR and PS6K was significantly higher in ASMCs after 2-h stimulation by IL-8 (20 ng/ml) than in PBS control samples (Figure 5A). We then treated ASMCs with PtCM, ASMC or fibrocyte condition medium, or PBS control for 2 h (Figure 5B). The mRNA expression of mTOR was significantly higher in ASMCs stimulated using PtCM than in PBS control samples. We also observed increases in PS6K expression in ASMCs following stimulation using PtCM (increasing trend; *p* = 0.09).

### Condition Medium From Patient Co-cultures Promoted AKT Phosphorylation in Airway Smooth Muscle Cells and Interleukin-8 Promoted PARS40 Phosphorylation in Airway Smooth Muscle Cells

**FIGURE 6:**
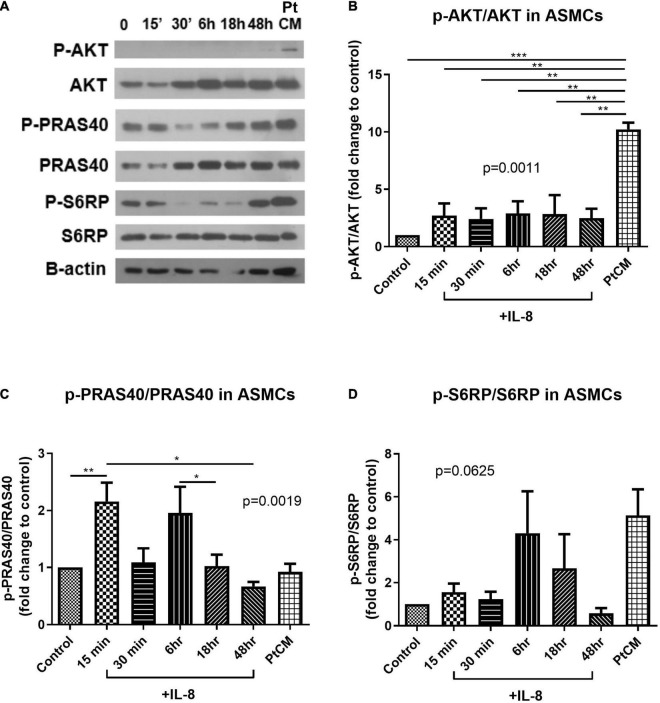
AKT-PRAS40-mTOR in ASMCs co-cultured with IL-8 or condition medium. **(A)** ASMCs were stimulated using IL-8 (20 ng/ml) at various time points: 15′, 30′, 6, 18, and 48 h or condition medium from ASMC co-cultured with FCs from patients (Pt CM, 3:1 dilution with culture medium. 6 h treatment). **(B)** Phosphorylation status of AKT, **(C)** PRAS40 and **(D)** ribosomal protein S6 (S6RP) in ASMCs following stimulation by IL-8 or PtCM (*n* = 5∼6). *P < 0.05; **P < 0.01; ***P < 0.001.

Western blot analysis was used to test the active status of AKT/mTOR signaling in ASMCs following IL-8 stimulation (for various durations) or PtCM (for 6 h) (Figure 6A). PtCM was shown to promote the phosphorylation of AKT in ASMCs (Figure 6B). IL-8 was shown to promote the phosphorylation of PRAS40 after 15-min stimulation (Figure 6C). We observed an increase in the phosphorylation of S6RP in ASMCs after stimulation using IL-8 or PtCM for 6 h (Figure 6D).

## Discussion

Co-culturing ASMCs with fibrocytes from asthmatic patients was shown to increase IL-8 expression in supernatant and IL-33 expression in ASMCs, compared to culturing ASMCs alone. Note that co-culturing ASMCs with fibrocytes from normal subjects had no effect. The effect of corticosteroid on IL-8 production was less pronounced in ASMCs co-cultured with fibrocytes from asthmatic patients than in ASMCs co-cultured from normal subjects. Treating ASMCs with the condition medium of co-culture from asthmatic patients or IL-8 was shown to induce the mRNA expression of mTOR and its substrate PS6K in ASMCs. Treating ASMCs with the condition medium of co-culture from asthmatic patients was shown to increase the phosphorylation of AKT and mTOR downstream signaling S6RP in ASMCs. Treating ASMCs with IL-8 alone was shown the enhance the phosphorylation of PRAS40 in ASMCs. Our work provides novel findings pertaining to the effects of corticosteroids and the proliferative signaling of AKT-PRAS40-mTOR in ASMCs co-cultured with fibrocytes from asthma patients.

IL-8 in the airways of individuals with neutrophilic asthma responds poorly to corticosteroids (21, 22). There are a limited number of treatment option specific to neutrophilic asthma, and the mechanism underlying IL-8 production in such cases is crucial to the development of new treatment regimes. Our observation of increased IL-8 expression with lower corticosteroid-induced IL-8 suppression suggests that fibrocytes have a paracrine effect on ASMCs in asthmatic patients (Figures 2A, 3A). Our results are comparable to those obtained in a similar previous study (14), which reported the involvement of NF-κB and ERK1/2 signaling in IL-8 production. In future studies, it would be interesting to explore the cell source of IL-8 in co-culture and signaling involved in the less corticosteroid suppression on IL-8 production.

In Figure 3A, we showed the reduced level of IL-8 in the ASMCs co-cultured with fibrocytes from asthma after steroid treatment by comparison to the vehicle treatment. The vehicle and the steroid treatments were done in the same co-culture experiment from the same subject. The result showed the level of IL-8 of co-culture with steroid treatment was lower than co-culture without steroid treatment (their own control) with statistically significant despite higher IL-8 production in co-culture of asthma patients and lower IL-8 production in that of normal subjects (Co-culture vs. ASMCs alone, Figure 2A). By showing the suppression effect on IL-8 occurred only at the highest concentration of steroid in co-culture of asthma patients, compared with the suppression at relative lower concentration of steroid in that of normal subjects (Figure 3A), and the pattern of IL-33 suppression by steroid was no different between asthma patients or normal subjects (Figure 3C), which was also higher IL-33 production in co-culture of asthma than that of normal subjects (Co-culture vs. ASMC alone, Figure 2C), we could tell the different steroid suppressive effects on IL-8 production between co-culture of asthma and normal subjects. However, the reduced effect of steroids on IL-8 secretion in ASMCs co-cultured with fibrocytes from asthmatics cannot be totally excluded the effects of increased secretion of IL-8 in co-culture of asthma. Further ELISA with actual concentration and more subject numbers are required for explore the steroid suppressive effect on high concentration IL-8 production in co-culture of asthma. However, the present study demonstrated the different pathological effects in co-culture between asthma patients and normal subjects.

Our observation of increased IL-33 in the lysate of ASMCs co-cultured with fibrocytes of asthmatic patients has not previously been reported (Figure 2C). Researchers have previously reported that increased IL-33 expression in ASM bundles of asthmatic airway is correlated with airway hyperresponsiveness (23, 24). In the current study, we discovered a potential source of IL-33 in asthmatic airways resulting from an interaction between ASMCs and fibrocytes. Unlike the current study, researchers previously reported that corticosteroids did not suppress IL-33 transcription in TNF-α stimulated ASMC cultures (24), due perhaps to differences in experiment design, such as the source of ASMCs and disease severity.

Signaling screen arrays revealed high PARS40 phosphorylation levels in ASMCs incubated with PtCM or IL-8 (Figure 4). PARS40 is a AKT substrate and key regulatory at the crossover point of AKT–mTOR transduction pathways (19). PRAS40 has been shown to inhibit the activity of the mTOR complex 1 (mTORC1) by preventing its binding to PS6K and 4E-BP1 (25). Active PS6K also phosphorylates S6RP to promote protein synthesis (26). Studies have shown that AKT is able to phosphorylate PRAS40 directly, resulting in the dissociation of PRAS40 from mTORC1 and the activation of mTOR function to promote cell growth (19, 27). It also has been shown that IL-8 can promote AKT-mTOR signaling pathways in a tissue specific manner (28). For example, IL-8 has been shown to promote the proliferation of prostate cancer through the activation of AKT-mTOR, resulting in the downstream phosphorylation of 4E-BP1 and PS6K, which could be abrogated by rapamycin (29). Similar phenomena have been observed in breast cancer cells, neutrophils, and ASMCs (9, 30–32). Based on the fact that co-culturing with fibrocytes from asthma patients significantly increased IL-8 expression levels in the supernatant, it was reasonable to explore the AKT-PRAS40-mTOR signaling. The elevated mRNA expression of mTOR and PS6K in ASMCs after stimulation with PtCM or IL-8 confirmed the reasonableness of this line of inquiry and is comparable with another study of cell line (33) (Figure 5).

In western blot analysis, 6-h incubation with PtCM induced strong AKT phosphorylation in ASMCs (Figure 6B). We observed a trend of increased downstream S6RP phosphorylation (Figure 6D); however, we observed no difference in the phosphorylation of PRAS40 (Figure 6C). The reasons for this are no doubt multifactorial. First, PtCM was the mixture of IL-8, IL-6 and other factors unchecked. It is possible that unknown factors promoted effects downstream from mTOR under the complex interactions involved in this co-culture model. Therefore, the effect of IL-8 was different from that of Pt CM. Furthermore, we did not perform serial time-point experiments on PtCM stimulation as was done for IL-8 stimulation, due to the limited availability of condition medium. Specific testing for IL-8 stimulation revealed a significant increase in the phosphorylation of PRAS40 in ASMCs in as little as 15 min (Figure 6C), which may compensate for missing part of the PtCM stimulation experiment. Besides, the change of p-PRAS40 is different from that on S6RP. The activation of S6RP is the downstream signaling of mTOR after phosphorylated PRAS40 relieving the inhibitory constraint on mTORC1 activity. This can be one explanation for the earlier activation of PRAS40 (up to 15 min, Figure 6B) and the activation of S6RP occurred around 6 h or earlier time point we did not check (between 40 min and 6 h, Figure 6D).

**FIGURE 7:**
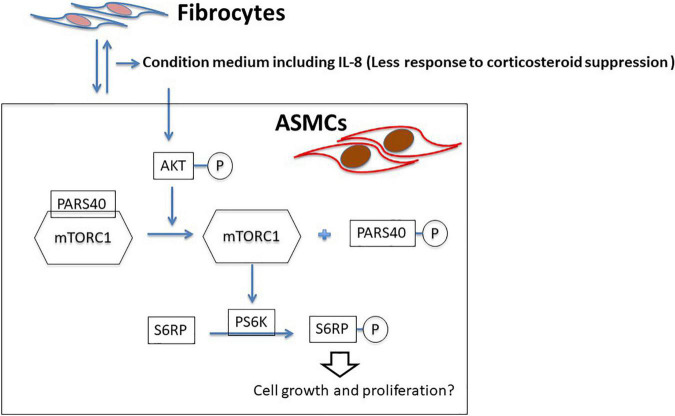
Proposed model of ASMC remodeling by fibrocytes in asthma patients via the axis of IL-8-AKT-PARS40-mTORC1. Interaction between fibrocytes and ASMCs increases the production of IL-8 in asthmatic airways, and corticosteroid-induced suppression of IL-8 production is reduced. IL-8 stimulation activating the axis of AKT-PARS40-mTORC1 in ASMC may contribute the downstream processes involved in ASMC proliferation/growth.

Taken together, we propose remodeling the IL-8-AKT-PARS40-mTOR axis in ASMCs interacting with fibrocytes in cases of asthma (Figure 7). It is possible that IL-8 production via ASMC-fibrocyte interaction initiates mTOR activation, resulting in the proliferation of ASMCs. Note that no previous studies have provided evidence of proliferation or hypertrophy in ASMCs co-cultured with fibrocytes (14, 15). This could perhaps be explained by the fact that *in vitro* co-culture models interfere mTOR with downstream signaling and/or ASMC proliferation.

The study has a number of limitations. First, we obtained CD34^+^ fibrocytes from patients; however, the ASMCs were purchased from a commercial supplier (i.e., they were not from the patients who provided the PBMCs). Note that simultaneous airway and blood sampling from the same subject and then growing a sufficient number of cells *in vitro* is challenging. Our use of commercial supply of cells made it possible to explore the underling molecular mechanism and consider further studies pertaining to the complex cellular interactions between FCs and ASMCs in cases of asthma. Second, we were constrained by the limited availability of FCs from patient blood, which limited the number of corresponding ASMCs for Quantitative Real-Time Reverse Transcription (rtPCR) and western blot experiments. mRNA level of some molecular markers, like IL-8, IL-6, or PRAS40 should be check in the future studies. Third, we also used PtCM to stimulate ASMCs instead of directly observing AKT-PRAS40-mTOR signaling in the ASMCs. Thus, the PtCM had to be diluted to cover the ASMC culture, which may have attenuated the effects. Nonetheless, even dilute PtCM promoted a significant increase in the expression of mTOR mRNA and p-AKT in ASMCs as well as increases in PS6K mRNA and p-S6RP expression. This suggests that PtCM has distinct biological effects on AKT-PRAS40-mTOR signaling in ASMCs.

## Conclusion

In conclusion, the interaction between ASMCs and fibrocytes from asthmatic patients was shown to increase IL-8 and IL-33 production. Corticosteroid-induced suppression of IL-8 production was less pronounced in the co-culture from asthma patients than from normal subjects. Corticosteroid-induced suppression of IL-8 production was more pronounced in samples co-cultured with fibrocytes from normal subjects than in samples co-cultured from asthmatic patients. Furthermore, IL-8 and conditioned medium co-cultured with fibrocytes from patients promoted AKT/mTOR signaling in ASMCs. Our results elucidate the novel role of fibrocytes and ASMCs in the process of inflammation and remodeling. These results may also inspire the development of novel treatments for asthma.

## Data Availability Statement

The raw data supporting the conclusions of this article will be made available by the authors, without undue reservation.

## Ethics Statement

The studies involving human participants were reviewed and approved by the Chang Gung Medical Foundation Institutional Review Board (No. 103 6844A3). The patients/participants provided their written informed consent to participate in this study.

## Author Contributions

T-YL: conceptualization, data curation, investigation, formal analysis, and writing—original draft. P-JC: data curation, resources, and formal analysis. C-YL, Y-LL, C-TY, S-ML, C-HK, and H-CL: data curation and resources. All authors contributed to the article and approved the submitted version.

## Conflict of Interest

The authors declare that the research was conducted in the absence of any commercial or financial relationships that could be construed as a potential conflict of interest.

## Publisher’s Note

All claims expressed in this article are solely those of the authors and do not necessarily represent those of their affiliated organizations, or those of the publisher, the editors and the reviewers. Any product that may be evaluated in this article, or claim that may be made by its manufacturer, is not guaranteed or endorsed by the publisher.

## Abbreviations

ASMCs: Airway smooth muscle cells
IL: Interleukin
mTOR: Mammalian target of rapamycin
PtCM: Condition medium from patients’ co-culture
GINA: Global Initiative for Asthma
PBMCs: Peripheral blood mononuclear cells
TSLP: Thymic stromal lymphopoietin
PS6K: P70S6 kinase
S6RP: S6 ribosomal protein
PRAS40: Proline-rich AKT substrate of 40 kDa.
