# Supplementary material for: Interaction Between CD34+ Fibrocytes and Airway Smooth Muscle Promotes IL-8 Production and Akt/PRAS40/mTOR Signaling in Asthma
Source: Front Med (Lausanne). 2022 Apr 25;9:823994. doi: 10.3389/fmed.2022.823994 (PMC9081978; doi:10.3389/fmed.2022.823994)
Supplement: Supplementary file 6 [file Data_Sheet_6.PDF]

[redacted]

CERTIFICATE OF ANALYSIS

Product Code: CC-2576
Product: BSMC-Bronch. Smooth Mus Cells, SmGM-2, cryo amp

Lot Number: 0000538849
Manufacture Date: 10-Apr-2016

TEST (Method)	SPECIFICATIONS Min.	Max.	Results
Tissue Acquisition Number	***	***	29661
DONOR CHARACTERISTICS			
Age	***	***	59 Y
Sex	***	***	MALE
Race	***	***	H
VIRUS TESTING			
HIV Test	***	***	Not Detected
HBV Test	***	***	Not Detected
HCV Test	***	***	Not Detected
MICROBIAL TESTING			
Sterility Test	***	***	Negative
Mycoplasma	***	***	Negative
CELL PERFORMANCE TESTING			
Cell Passage Frozen			3
Viability	>= 70%	***	96 %
Cell Count (Cells/ml)	>= 500,000	***	617000
Seeding Efficiency	>= 20%	***	52 %
Doubling Time (hours)	15	48	45 hrs
Alpha Actin Expression	Pass: >=30% positive	***	Pass

These cells were isolated from donated human tissue after obtaining permission for their use in research applications by informed consent or legal authorization. This product is for research use only. Details concerning the use of our cell and media products can be downloaded from our website at www.lonza.com/cell-protocols.

In addition to the specifications listed above, the following are guaranteed for all lots of this product using Lonza's Clonetics(TM) and Poietics(TM) Media, Reagents, and Protocols: Total Population Doublings >=15, Factor VIII Negative.

This lot has been reviewed by Quality Assurance in compliance with requirements of Lonza's Quality System.
This document was generated from a validated Part 11-compliant electronic system and thus handwritten signatures are not required.
